# A Case of Intramuscular Lipoma of Thenar Eminence With a Short Review of Published Hand Lipoma Case Reports

**DOI:** 10.7759/cureus.39628

**Published:** 2023-05-29

**Authors:** Aditya Soni, Milind Mehta, Kapil Shirodkar, Abhishek Singh, Gautam Talawadekar

**Affiliations:** 1 Trauma and Orthopaedics, Furness General Hospital, University Hospitals of Morecambe Bay NHS Foundation Trust, Barrow-in-Furness, GBR; 2 Radiology, Royal Lancaster Infirmary, University Hospitals of Morecambe Bay NHS Foundation Trust, Lancaster, GBR; 3 Trauma and Orthopaedics, Indira Gandhi Hospital, New Delhi, IND

**Keywords:** lipoma literature review, intramuscular lipoma, missed diagnosis, palmar lipoma, thenar lipoma

## Abstract

Lipoma is a common soft tissue tumour in the human body, but at the same time is very rare in the palm and rarer still in the thenar region. These lipomas in the hand can give rise to various problems such as cosmetic, functional, and neurological compromise among others and removing them becomes important when symptomatic. Diagnosing a hand pathology becomes important as a missed diagnosis can have long-term functional consequences for a patient. In the case report, we discuss a hand palmar prominence which presented as an effusion and later turned out to be a large lipoma. Further, we also present a literature review of published thenar lipoma cases to throw light on the nuances of this rare pathology location, which, to our knowledge, has not been done comprehensively.

## Introduction

The World Health Organization classifies soft tissue tumours into lipomatosis, lipomatosis of nerve, angiomyolipoma, lipoblastoma, myolipoma, chondroid lipoma, spindle cell lipoma, hibernoma, and benign simple lipoma [1]. Soft tissue lipoma is a combination of benign neoplasm and local hyperplasia of fat cells, with common locations being the back, shoulder, neck, and head with a common occurrence between the fourth and seventh decade of life. Further, the lipomas are subdivided into a superficial subtype, which lies in the subcutaneous tissue, and the deep subtype, which is under the dermis and investing deep fascia [2].

Lipomas in the hand are a rare occurrence, with thenar eminence subcutaneous region being the most common when it occurs. A subfascial lipoma (deep variety) can also occur in the hand but is generally pushed to the side due to the unyielding nature of aponeurosis [3]. We present a similar case of lipoma in the thenar eminence in a 32-year-old patient and discuss the clinical presentation, diagnostic workup, and surgical management of this patient’s palmar lipoma. Additionally, we singled out all the case reports of thenar lipoma published to date for a comprehensive literature review to throw light on the various peculiarities of such a pathology, which we believe will definitely help surgeons when they come across such cases.

## Case presentation

A 32-year-old, right-hand dominant white Caucasian male working as a manual labourer presented to the clinic with the main complaint of cosmetic disfigurement and functional impairment due to a palmar tumefaction in the left hand, which had been standing for a few months and progressively increasing. On examination, it was a painless prominence on the thenar eminence which felt soft with diffuse borders. There was no nerve involvement in the hand, verified by checking the dermatomes of the radial, median and ulnar nerves in the hand. There was reduced function due to a mass effect in terms of weak palmar grip and pinch.

**Figure 1:**
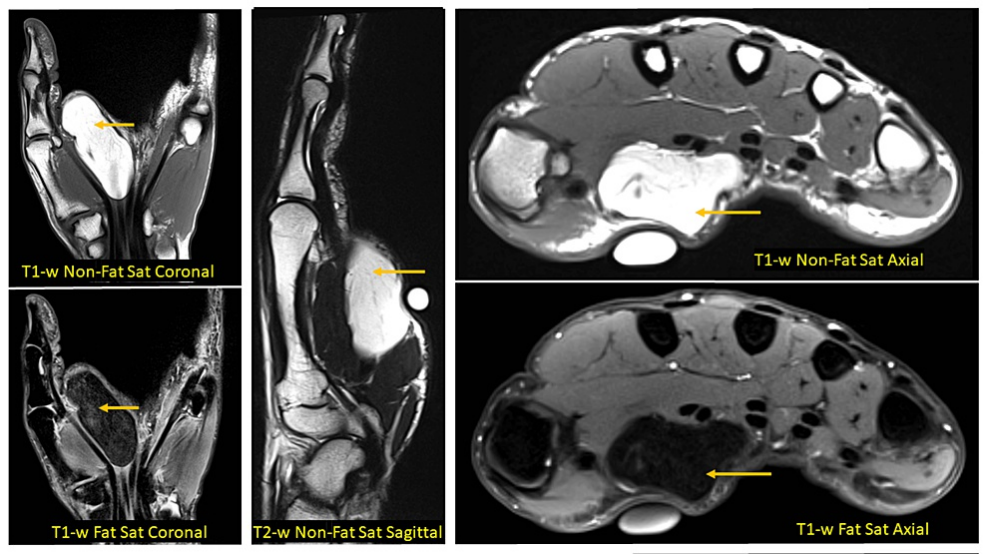
Magnetic resonance imaging findings: coronal, sagittal, and axial sections of the hand showing the mass among the thenar muscles.

It was labelled as an effusion or a ganglion in the first instance with further differentials of an infected cyst, soft tissue tumour, and foreign body granuloma. Magnetic resonance imaging (MRI) was obtained due to its higher sensitivity which showed a 5.8 × 2.5 × 1.8 cm lipoma within the flexor carpi radialis muscle (intramuscular) but separate from flexor digital tendons (Figure 1). The MRI showed homogenous fatty components appearing hyperintense on T1/T2 sequence and uniformly suppressing on short tau inversion recovery (STIR) images. There were no solid components or thick internal septations. Muscle fibres were not infiltrated with no associated effusion found. All the above features labelled the tumour as benign and a decision was made to excise it without opting for a biopsy.

**Figure 2:**
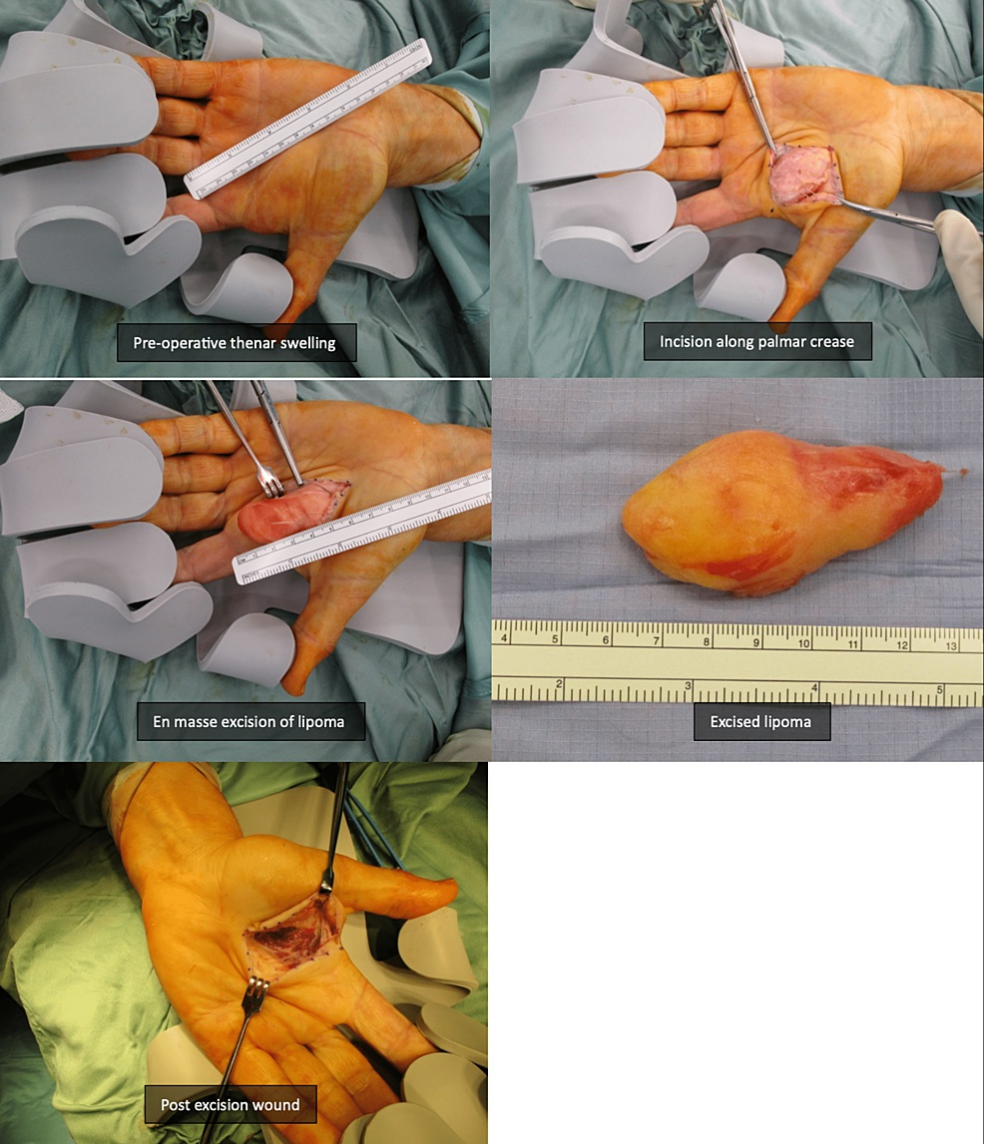
Intraoperative images.

It was excised uneventfully by an upper limb surgeon under a regional block. The lipoma was found to be arising from within the thenar muscles and was well encapsulated. Histological examination of the excised mass confirmed it to be a lipoma with benign characteristics. The surgical site healed well with full hand function retained by the patient, as checked by the recovered grip and pincer strength. The patient was followed up for six months and eventually discharged with no further recurrence or problems. Figure 2 shows the intraoperative and specimen images.

## Discussion

The first documented hand lipoma case report dates to 1971 when Phalen et al. published a report of 15 hand and wrist lipomas. They did not find any malignancy in any of the cases but almost 25% had a neurological compromise [4]. Another study also reported a similar number [5]. The palmar incidence of lipomas has been seen as high as 7.5% in the hand which is higher than the previous estimates of 1-4% [5-7].

The presence of thick unyielding palmar fascia is believed to cause the larger lipomas to present peripherally on the palm and along the carpal tunnel, and at the same time, the palm due to its unique anatomy and gap under the palmar fascia gives rise to deceptively larger lipomas than other sites [8,9]. While operating, the same anatomical configuration necessitates the need for more extensive dissection [10,11]. Some of the lipomas can be seen extending across from the dorsum to the palmar aspect of the hand and need a dual incision on the hand to completely remove them [8]. The lipomas have a similar histological and gross appearance irrespective of their location, and corroborating it a study exclusively examining intramuscular lipomas found no difference between the different sites of lipomas [12].

In the days when MRI was not widely prevalent, a study in 1991 found ultrasound a useful investigation for delineating lipomas from other tumours and foreign bodies. The study concluded that an experienced sonographer was the right person to perform these studies for the results to be reproducible and reliable [13]. Later, starting with a case series in 1997, MRI was found to be a more sensitive, accurate, and useful investigation for hand lipomas [14]. MRI showed the extent and involvement of surrounding structures, and it helped plan surgery better in multiple studies [8,12,15-23]. Moreover, in the recent past, ultrasonography was not found sensitive enough in deeper lipomas/encapsulated lipomas as it missed the bilobed structure as well as the intramuscular extensions [24,25]. The relations become important to know while excision, more so if a malignancy is suspected. In most cases, MRI imaging can delineate the diagnosis with good accuracy but in cases of radiological uncertainty, an image-guided biopsy has been recommended by Cribb et al. [15]. Interestingly, there were cases where the MRI showed a malignant potential but was completely ruled out after biopsy [10].

Surgical excision is warranted and should be offered if the lipoma is greater than 5 cm, has a functional restriction, muscle dysfunction, reduced strength, triggering, pain, or paresthesia [18]. A lipoma can be conservatively managed if it is not a cosmetic or above-mentioned problem. Even in cases of conservative management, a needle biopsy should be performed at the very least if there are concerns about malignancy [19]. Traditionally, marginal excision has been the treatment of choice but a wider radical excision needs to be planned if there are signs of malignancy on MRI or needle biopsy [8,9,15,18,20]. The lipoma generally comes out en masse due to the thin fibrous capsular covering around it, but failure to remove it completely is known to cause recurrences [24]. The rarer cases when the tumour is not very well encapsulated can also be treated with marginal excision but need longer follow-ups of up to five years [8,26]. Similarly, longer follow-ups are needed with cellular atypical features on biopsy after excision [27]. In cases of neurological compromise of hand nerves, a diagnosis of lipoma should be kept as a differential if no obvious cause is evident [8].

There is a suggestion that a trained hand surgeon operates on benign cases, and in cases of suspicion of malignancy, a cancer surgeon along with a hand surgeon should be present [9,15,26]. In a few of the published studies, a size greater than 5 cm (giant lipoma) is to be considered to have malignant potential, unless proven otherwise, along with the need for greater vigilance in the postoperative period [9,28]. Giant benign lipomas of the hand in one of the published studies were shown to mimic well-differentiated liposarcomas on MRI and required immunohistochemical tests to differentiate the chromosomal structural disorders in the lipoma specimen [28]. Both benign and malignant tumours are known to have a mutation around the chromosomal region 12q13-15. While the benign version has a simple anomaly of inversion or translocation, the malignant tumours have a giant chromosome or a supernumerary ring [28]. Consequently, an MRI and needle biopsy followed by cytogenetic/histological testing is useful in such cases [29]. Understandably, a referral to the national sarcoma service is warranted in proven malignant cases after needle biopsy/excision [5,30].

Interestingly, one case report from 2007 and a publication from 1972 mentioned that the lipoma was seen to erode the metacarpal, which is an unusual presentation, as lipomas do not affect the bone; further, there was full recovery post-excision [17,31]. Apart from the recurrence that can occur due to incomplete removal, hematoma formation has also been described post-surgery due to the large dead space being left [32]. This requires close follow-up in patients on antiplatelet and blood thinning medications [29]. Lipomas in the hand have a very real potential to cause neurological compromise, especially of the thumb due to compression of the radial digital nerve, more so by giant lipomas measuring over 5 cm in size [12,33].

The first line of investigation in such cases is usually an ultrasound due to ready availability but is not immune to interoperator variability and difficulty in defining borders; hence, the challenge of differentiating it from other soft tissue masses [34]. Where possible, MRI is the preferred modality to accurately define and diagnose lipomas. Knowing the extent becomes crucial while excising to avoid damage to tendons and neurovascular structures in the hand. Intermuscular lipomas can cause problems with muscle function as well due to pressure in encapsulated type and pressure plus invasion in the infiltrating type [35,36]. Understandably, this requires longer muscle strengthening, and knowing this also helps in managing patient expectations. Interestingly, even with the above problems, pain is a late symptom [36].

Going through the published literature on palmar and specifically thenar lipomas, it was found that only a handful of thenar lipomas have been published (Table 1). Among the published cases concerning thenar lipomas, 12% had nerve compromise while only 4% had tendon involvement. Notably, mid-palmar lipomas show a 45% incidence of neural compromise, which is more than three times that in thenar lipomas. The largest thenar lipoma among the reported studies was 8.1 × 4.9 × 6.7 cm [29].

**Table 1:** Summary of published thenar lipoma case reports NS = not specified; USG = ultrasonography; MRI = magnetic resonance imaging

Study	Age (years)	Gender	Size (cm)	Duration to presentation	Presenting complaints	Neurological compromise	Investigations done
Phalen et al. 1971 [4]	62	F	4 × 3 × 1.5	3 days	Cosmetic disfigurement	None	NS
Oster et al. 1989 [8]	50	M	8 × 5 × 2	NS	Reduced function	None	NS
Höglund et al. 1991 [13]	NS		NS	NS	NS	None	USG + MRI
Goodman et al. 1997 [14]	51	F	NS	6 months	Cosmetic disfigurement	None	MRI
Lee et al. 2004 [12]	56	F	4.5 × 3 × 3	1 year	Reduced function	Yes	MRI
	45	F	3 × 2.5 × 2	4 years	Reduced function	None	MRI
	52	F	7 × 4.5 × 4	3 years	Reduced function	Yes	MRI
	68	F	6 × 5 × 4	4 years	Reduced function	None	MRI
Inaparthy et al. 2005 [37]	65	F	8 × 4 × 3	8 years, enlarging for the last 18 months	Cosmetic disfigurement	Yes	MRI
Boussouga et al. 2006 [16]	40	F	NS	NS	Reduced function	Yes	USG + MRI
Kamath et al. 2006 [9]	38	M	6.5 × 4	18 months	Cosmetic disfigurement	None	MRI
Schoffl et al. 2007 [17]	47	F	NS	NS	Reduced function	None	MRI
Grivas et al. 2008 [28]	60	F	4.5 × 4.5 × 3	1 year	Reduced function	Yes	MRI
Nadar et al. 2010 [30]	63	M	4.5 × 2.5 × 1.2	14 months	Reduced function	Yes	MRI
Chatterton et al. 2013 [38]	67	F	8 × 6 × 3	2 years	Reduced function	None	USG + MRI
Chernev et al. 2013 [25]	87	F	3.8 × 2.2 × 1.5	Few months	Discomfort/Pain	None	MRI
Yadav et al. 2013 [18]	61	F	9.5 × 4.5 × 4	5 years	Reduced function	None	MRI
Ferrando et al. 2014 [19]	72	M	2.3 × 2.2 × 1.4	NS	Reduced function	None	USG + MRI
	60	F	6 × 5 × 3	NS	Reduced function	None	USG + MRI
Iyengar et al. 2014 [20]	68	F	5.9 × 3.2 × 2.2	2 years	Reduced function	None	MRI
Leclere et al. 2015 [39]	63	M	0.6 × 0.4 × 0.4	NS	Cosmetic disfigurement	Yes	MRI
	56	M	6 × 4 × 2	NS	Discomfort/Pain	None	MRI
Papakostas et al. 2015 [24]	59	M	4.9 x 3.7 x 2.9	10 years, elarging for the last 2 years	Reduced function	Yes	MRI
Raposo et al. 2015 [21]	62	F	3.5 × 2.5 × 1.4	1 years	Reduced function	Yes	USG + MRI
Yildiran et al. 2015 [26]	49	F	5 × 3 × 2	NS	Discomfort/Pain	Yes	MRI
Al-Qattan et al. 2016 [27]	57	F	6 × 7	NS	NS	None	MRI
	48	M	4 × 5	NS	NS	None	MRI
	53	F	5 × 5	NS	NS	None	MRI
Ergun et al. 2016 [10]	NS	NS	NS	NS	NS	None	MRI
Venkatesh et al. 2016 [22]	54	F	17 × 10	NS	Cosmetic disfigurement	None	MRI
Cemboluk et al. 2017 [23]	56	F	2.5 × 5.9 × 6.2	2 years	Reduced function	Yes	MRI
Marteau et al. 2019 [5]	43	F	5.5 × 4.5 × 1.4	8 months	Cosmetic disfigurement	None	MRI
	72	M	3.5 × 3 × 2.5	3 years	Cosmetic disfigurement	None	MRI
	64	F	5 × 2.5 × 1	5 years	Cosmetic disfigurement	None	MRI
	55	F	3.7 × 2.7 × 2.4	2 years	NS	NS	MRI
Balvis et al. 2020 [32]	NS	NS	NS	NS	NS	None	MRI
Ulaganathan et al. 2020 [29]	65	F	8.1 × 4.9 × 6.7	25 years	Reduced function	None	MRI

## Conclusions

Palmar lipomas are a very rare entity and very few have been reported in the past. Even rarer are lipomas in the thenar eminence, as in this case. Missing a diagnosis in the palm can have serious complications with hand function, as lipomas in the palm can deceptively present with clinical features intermingling with other lesions. There was no associated pain but only cosmetic and functional impairment, with MRI being the investigation of choice to diagnose and determine the type and relations to safely excise the lesion. Surgical excision followed by histologic analysis was the final management which led to good recovery and full function. Reviewing the previously reported thenar lipoma cases gives us a good insight into the different patterns of presentation and factors to keep in mind while managing them.
